# Masking important information to assess the robustness of a multimodal classifier for emotion recognition

**DOI:** 10.3389/frai.2023.1091443

**Published:** 2023-03-22

**Authors:** Dror Cohen, Ido Rosenberger, Moshe Butman, Kfir Bar

**Affiliations:** Faculty of Computer Science, The College of Management Academic Studies, Rishon LeZion, Israel

**Keywords:** emotion-recognition, multimodal, perturbation, text-audio, NLP

## Abstract

Deep neural networks have been proven effective in classifying human interactions into emotions, especially by encoding multiple input modalities. In this work, we assess the robustness of a transformer-based multimodal audio-text classifier for emotion recognition, by perturbing the input at inference time using attacks which we design specifically to corrupt information deemed important for emotion recognition. To measure the impact of the attacks on the classifier, we compare between the accuracy of the classifier on the perturbed input and on the original, unperturbed input. Our results show that the multimodal classifier is more resilient to perturbation attacks than the equivalent unimodal classifiers, suggesting that the two modalities are encoded in a way that allows the classifier to benefit from one modality even when the other one is slightly damaged.

## 1. Introduction

Automatic emotion recognition is among the most widely studied applications of machine learning. People express their emotions through voice, facial expressions, hand gestures, body movements, writing, arts, and more. In this work, we focus on speech and its transcriptions.

In the last few decades, there have been a considerable amount of research in the area of emotion recognition. Before the era of deep learning, most of the algorithms for emotion recognition were developed based on some traditional machine-learning techniques, such as support vector machines (SVM). However, recent developments use deep neural networks, which have shown significant performance improvement in emotion recognition (Fathallah et al., 2017; Mirsamadi et al., 2017; Sapiński et al., 2019).

Most previous studies focus on emotion recognition using only one modality, such as text, video, or voice. However, it has already been shown that algorithms for emotion recognition that are based on multiple modalities, perform better than the ones that use only one modality (Povolny et al., 2016; Krishna and Patil, 2020). In our study, we go beyond this comparison as we measure the contribution of each encoded modality by intentionally perturbing the input at inference time to either exclude or corrupt some intelligible information, which has already been proven effective for emotion-recognition classification, and monitoring changes in the performance of the model. We use two modalities, voice speech and its transcription. For comparison, we train two unimodal classifiers, one that uses only the acoustic speech signal, and one that uses only its textual transcriptions, and examine their performance under equivalent conditions.

It has been shown (Bolinger and Bolinger, 1986; Shintel et al., 2006) that prosody is an important aspect of emotional expression through speech, and that vocalizations with high fundamental frequency (*F*0), also known as voice pitch, and high intensity are associated with positive emotion expressions and high arousal; on the other hand, low-pitch vocalizations may indicate negative emotions and low arousal. Moreover, there is evidence that people generate iconic vocalizations to express different meanings (Perlman and Cain, 2014). Specifically, Perlman et al. (2014) have shown that different vocalizations—characterized by average voice pitch, pitch range, duration, intensity, and harmonics-to-noise ratio—were used to differentiate between antonymic pairs of adjectives and adverbs. Additionally, there is a large body of work that studies how voice pitch is associated with everyday social interactions. For example, Pisanski et al. (2018) showed that human-voice modulation in non-verbal communication, elicits favorable judgements and behaviors from potential mates.

In this study, we focus on measuring the robustness of a multimodal audio-text emotion-recognition model, by performing perturbation attacks designed to hide or modify information deemed important for emotion recognition. The multimodal classifier is evaluated on both, the perturbed and unperturbed inputs, for which we measure classification performance. The difference in performance is used to measure the impact of the specific attack on the examined model. Concretely, inspired by the studies mentioned above that indicate adjectives, adverbs and pitch as important components for expressing emotions, on the text side we hide words from the input that belong to a specific part-of-speech category. We focus on adjectives and nouns, leaving adverbs out of scope due to their relatively low frequency. On the audio side, we increase or decrease the voice pitch by half a tone.

In a recent review paper (Liang et al., 2022) on multimodal machine learning, the authors have identified six core research challenges related to multimodal analysis: representation, alignment, reasoning, generation, transference, and quantification. We believe that in this study, we make contributions to the alignment challenge, which is related to the connections and interactions between the modalities. We study the interaction between some specific part-of-speech categories and the audio modality, as well as the interactions between the pitch and the text modalities.

Therefore, our research focuses on the following research questions:

RQ1. Does combining speech transcription with voice improve performance of an emotion-recognition classifier?

RQ2. Is the multimodal emotion-recognition classifier more resilient to part-of-speech perturbation attacks than the equivalent text-only classifier?

RQ3. Is the multimodal emotion-recognition classifier more resilient to pitch-modulation perturbation attacks than the equivalent audio-only classifier?

We encode each modality using a transformer-based architecture; for the speech signal we use wav2vec 2.0 (Baevski et al., 2020), and for the transcriptions we use BERT (Devlin et al., 2019). In our multimodal architecture design we lean on the model-level fusion paradigm as we combine the last part of the two unimodal models, one for voice and one for text. To train and evaluate our models, we use the Interactive Emotional Dyadic Motion Capture (IEMOCAP) dataset (Busso et al., 2008), one of the largest open-sourced multimodal resources available for emotion recognition. It consists of approximately 12 h of filmed conversations, broken down into speech, text transcription, video, and motion capture of face. The conversations are manually annotated into categorical emotion labels, which can be used in supervised classification settings.

Our results suggest that a multimodal model for emotion recognition is more resilient to the perturbations we perform on to the input, than the equivalent unimodal models.

## 2. Related work

Emotion recognition has been widely studied over the years, and different modalities have been evaluated, including audio, text, facial expressions, and body movements. Some works have already shown that combining several modalities improves classification performance for emotion recognition. The two main dimensions on which multimodal classifiers differ from each other are (1) the modalities which they encode, and (2) the approach that they take to combine the modalities together, also known as fusion strategy. Typically, a multimodal architecture is designed either by concatenating the features extracted from each modality, known as feature-level fusion strategy, or by concatenating the output of unimodal models that encode and classify each modality independently. The latter is divided into three known strategies: decision-level fusion in which the final output of the classifiers are combined and used as an input for a mathematical formula to generate the final prediction; score-level fusion, which is almost the same as decision-level fusion, but instead of using the final output of the unimodal classifiers, their underlying label distributions are used; and finally, model-level fusion, in which some output layers of the unimodal models are concatenated and extended with a classification component. Our model is designed based on the latter strategy.

There is a relatively large number of studies on emotion recognition that focus on audio and video. Busso et al. (2004) built a relatively small collection of recordings of an actress expressing four basic emotions (sadness, happiness, anger, and the neutral state) through reading sentences in front of a camera. They recorded her facial expressions and voice, and used it to train a multimodal classifier that classifies a given recording into the four emotion labels. They experimented with two fusion strategies, decision-level and model-level, and showed that the two perform similarly, but much higher than unimodal classifiers. Each recording was divided into three modalities: audio, face video, and body video. They proposed a deep belief network that combines all modalities together, attaining better results than some baseline models, which use either a more traditional classification algorithm, such as support vector machines (SVM), or encode less modalities. In another work on audio-video emotion recognition (Zhang et al., 2017), the authors developed a deep belief network that fuses audio and video into a single classification model. Schoneveld et al. (2021) proposed a deep neural network that encodes audio and video for emotion recognition. Their proposed architecture follows the model-level fusion strategy in which the two unimodal models are combined using a deep feed-forward network, which they train end to end. As opposed to all other previous work mentioned above, Schoneveld et al. (2021) worked on a dimensional view of emotions, in which emotions are embedded on a continuous 3 dimensional space with axes represent the valence, arousal, and potentially the dominance of the emotion. The other representation is the categorical view in which emotions are represented as discrete states. Schoneveld et al. (2021) reported on new state-of-the-art results for valence detection on the RECOLA dataset (Ringeval et al., 2013), using their multimodal setup.

Some works use more than two modalities. For example, Poria et al. (2016) proposed a multimodal classifier that encodes speech, voice tone, and facial expressions for emotion recognition, using a temporal convolutional neural network. Tripathi et al. (2018) introduced a simple architecture that concatenates three modalities—speech, transcriptions, and motion capture—and reported on a significant improvement over the equivalent unimodal classifiers. Earlier that year, a similar concatenation approach was suggested by Poria et al. (2018), who also showed that a multimodal classifier outperforms models that use single modalities for emotion recognition.

In our study, we design a model that encodes speech voice and its transcription for emotion recognition. Those two modalities have been the main focus of a recent review article (Atmaja et al., 2022), which surveys a large number of works on speech emotion recognition. To name a few, Cho et al. (2019) proposed a neural architecture for emotion recognition using speech voice and its transcription, and achieved a significant improvement of 24% in accuracy, over the equivalent unimodal baselines. Atmaja and Akagi (2020) took a multitask learning approach to encode some acoustic features extracted from voice, as well as embeddings of words from the transcriptions, and reported on a significant improvement in valence prediction over a unimodal classifier that uses only acoustic features. Several other works (Cai et al., 2019; Atmaja et al., 2020; Chen and Zhao, 2020; Liu et al., 2022) that employ different fusion strategies, as well as different model architectures, have clearly shown performance improvements achieved by a multimodal architecture over unimodal baselines, for categorical emotion recognition.

In this work, we study the robustness of a multimodal classifier that encodes voice speech and its transcription, by perturbing the input in different ways, specifically designed to make impact on emotion recognition, and examining the effect of such perturbation attacks on the performance of the model. In a recent work (Schiappa et al., 2022), unrelated directly to emotion recognition, the authors described a similar perturbation methodology which they use to study the robustness of a multimodal classifier that encodes video and text. They have tested various real-world perturbation strategies and measured how significantly they impact the performance of the model. Among the strategies the authors have tested, there is one in which they replaced different part-of-speech categories with a placeholder. They concluded that models are generally more robust when only the text gets perturbed, as opposed to when only video was perturbed. The robustness of a multimodal neural network has been studied also by Yang et al. (2021), who even proposed a way to combine the two modalities in a neural architecture that have better resilience to such perturbation attacks performed only on one source.

Similarly, our work focuses on the robustness of a multimodal audio-text neural network for emotion recognition. We perturb the input, each modality at a time, and examine the change in performance. Our perturbation techniques are specifically designed to corrupt information that has been proven effective for emotion recognition.

## 3. Data

We work with the Interactive Dyadic Emotional Motion Capture Database (IEMOCAP) (Busso et al., 2008) which was released in 2008. IEMOCAP is a collection of recordings of five dyadic sessions of different pairs of actors, one female and one male. The actors were given two assignments: (1) They were instructed to play three written scripts, which were designed to generate emotions. (2) The actors were given a general scene description, on which they were asked to improvise a conversation. Each conversation is provided along with a transcription, a speech audio file, a video file, and some motion-capture data. Overall, the dataset contains approximately 12 h of recordings. The conversations were manually divided into utterances of length between 3 and 15 s which were labeled by at least three human annotators for emotion labels. The following ten labels were used: neutral, happiness, sadness, anger, surprise, fear, disgust, frustration, excited, and other. Table 1 summarizes the label breakdown of the utterances included in each session. The “xxx” label refers to utterances for which the annotators have not come into agreement on label assignment; it comprises about 25% of the dataset, which we decided to exclude from our experiments.

**Table 1:** IEMOCAP label breakdown per session.

**Label**	**Ses. 1**	**Ses. 2**	**Ses. 3**	**Ses. 4**	**Ses. 5**	**Total**	
Neutral	384	362	320	258	384	1,708	(17.0%)
xxx	415	436	572	564	520	2,507	(24.9%)
Frustrated	280	325	382	481	381	1,849	(18.4%)
Angry	229	137	240	327	170	1,103	(10.9%)
Sad	194	197	305	143	245	1,084	(10.7%)
Happy	135	117	135	65	143	595	(5.9%)
Excited	143	210	151	238	299	1,041	(10.3%)
Surprised	25	17	28	19	18	107	(1.0%)
Other	1	1	0	1	0	3	(0.0%)
Fear	12	9	2	7	10	40	(0.4%)
Disgust	1	0	1	0	0	2	(0.0%)

As shown in the table, some labels are less frequent than the others. Consequently, in this study we decide to work only with utterances annotated with the following five labels: sad, anger, frustrated, happy, and excited. Following common practices (Poria et al., 2016; Neumann and Vu, 2019; Sutherland et al., 2021; Schmitz et al., 2022), we merge all happy and excited instances into a single label, resulting in a dataset of utterances that are labeled with four labels, as summarized in Table 2.

**Table 2:** Label breakdown per session in the filtered dataset.

**Label**	**Short**	**Ses. 1**	**Ses. 2**	**Ses. 3**	**Ses. 4**	**Ses. 5**	**Total**	
Frustrated	fru	280	325	382	481	381	1,849	(32.5%)
Angry	ang	229	137	240	327	170	1,103	(19.4%)
Sad	sad	194	197	305	143	245	1,084	(19.1%)
Happy+Excited	hap	278	327	286	303	442	1,636	(28.8%)

We define a short caption for each label in the *Short* column, which we use throughout the paper.

In our work, each utterance is represented by a voice speech file and its textual transcription. The audio files were down-sampled to 16 kHz, and the texts were taken as is.

## 4. Methodology

**Figure 1:**
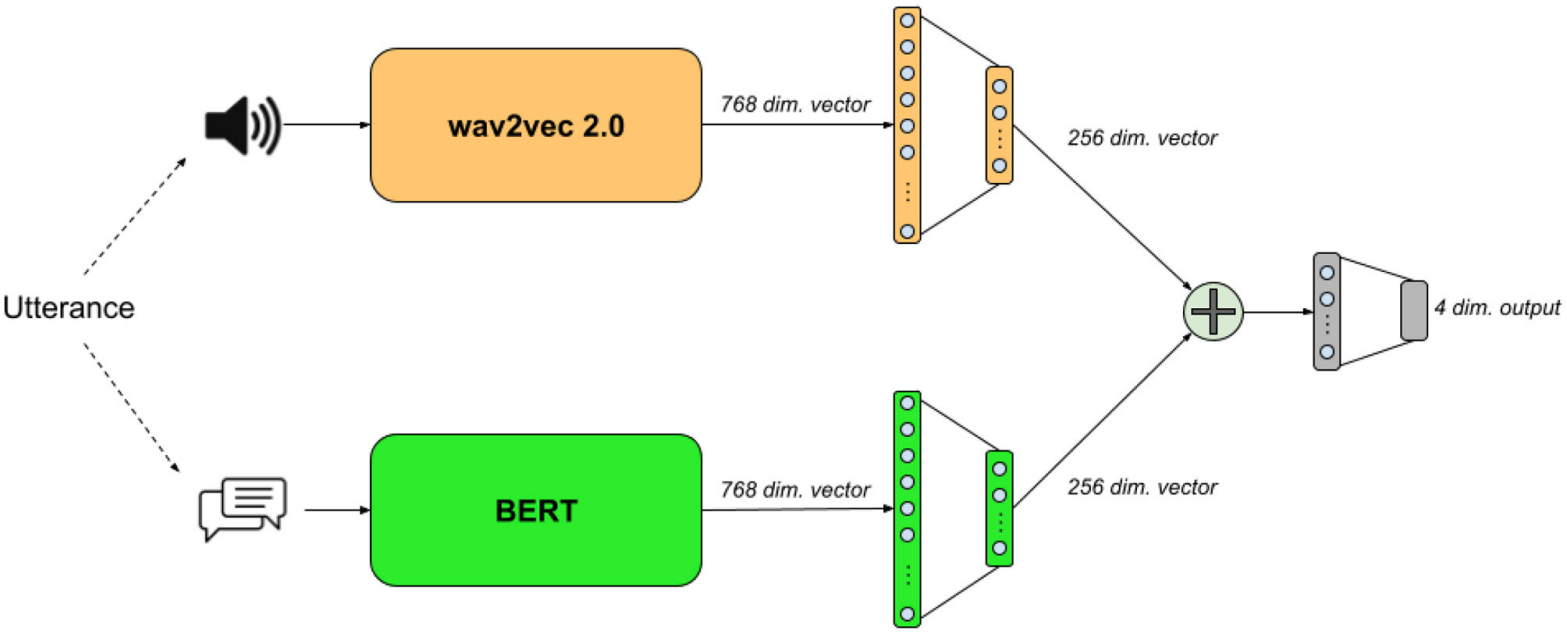
The text+audio model's architecture.

We design a relatively simple neural architecture for multimodal classification comprises two encoders, one for each modality, which works on the utterance level. The two encoders produce an encoded version of the voice speech (audio) and its transcription (text), which we concatenate into as a single representation used for classification. Figure 1 visualizes our multimodal network architecture. To compare the performance of our multimodal architecture, we use the same unimodal encoders individually for classification. We elaborate further on each encoder in the following three sections.

### 4.1. The text encoder

Our text encoder is based on BERT (Devlin et al., 2019), a common bidirectional transformer (Vaswani et al., 2017) encoder. Following standard practices, we apply WordPiece (Schuster and Nakajima, 2012) tokenization on the input transcriptions, place a [CLS] token in front of the sequence of tokens, and close it with a final [SEP] token. We use the *base*^1^ version of BERT which generates an output vector of 768 dimensions for every input token. We take only the output vector of the [CLS] token, which was originally designed for classification tasks, and feed it into a feed-forward component made of two ReLU-activated fully-connected layers with 512 and 256 neurons, respectively.

The text encoder is used in two classification settings, unimodal and multimodal. For unimodal classification, the final 256-dimensional vector is fed into a final classification layer, activated by softmax, which generates a distribution over the four emotion labels. The multimodal classifier is described in the following section. To train the unimodal classifier we use a batch size of 16, a learning rate of 10^−6^, and a weighted cross-entropy loss function, with weights proportional to the number of instances of each label. We use early stopping with a patience value of 10.

### 4.2. The audio encoder

We design the architecture of the audio encoder in a similar way to the one presented by Pepino et al. (2021). We start by processing the raw waveform of the voice speech using wav2vec 2.0 (Baevski et al., 2020), and feed its 768-dimensional mean-pooled output vector into a feed-forward network composed of two layers with 512 and 256 neurons, respectively. Each layer is activated by ReLU and extended by a dropout function with drop probability of 0.2.

The input sequence length was set intentionally to 246, 000, which is the static sequence length of wav2vec 2.0. Along with a sample rate of 16, 000, our input length corresponds to 15.375 s. Input sequences shorter than this length are padded accordingly. For longer sequences we use only the first 246, 000 samples, and cut out all samples that follow. Overall, there are only 1.16% instances that are longer than the 246, 000 limit.

Similar to the text encoder, we use the audio encoder for both unimodal and multimodal classification. The multimodal classifier is described in the following section. In unimodal classification, the final 256-dimensional vector is fed into a final classification layer activated by softmax, which generates a distribution over the four emotion labels.

To train the unimodal classifier, we use batch size of 8, a learning rate value of 10^−6^, and a weighted cross-entropy loss function, with weights proportional to the number of instances of each label. Same as before, we use early stopping with patience of 10.

### 4.3. The multimodal classifier

The multimodal classifier is composed of the two encoders, whose 256-dimensional output vectors get concatenated into a final 512-dimensional vector, which we feed into a ReLU-activated fully-connected layer with 128 neurons. Finally, we feed the 128-dimensional vector into a final classification layer activated by softmax to generate classification probabilities for the four emotion labels.

We use a batch size of 4, since the input for the multimodal classifier takes more memory than the input for the unimodal classifiers. Additionally, we use a learning rate of 5 × 10^−6^ with a weighted cross-entropy loss function same as described above.

### 4.4. Experimental settings

We fine-tune the three classifiers, text, audio, and multimodal, on the five sessions of the IEMOCAP dataset, and evaluate their performance. Each utterance, represented by speech and its textual transcription, is used as a single classification instance. We perturb each utterance, by hiding or modifying some information deemed important for emotion recognition. We train and evaluate the models under each perturbation attack individually, and measure their performance. Embracing common practices, we evaluate our models on a five-fold cross-validation setting in which we exclude one full session from the training set, and use it for evaluation. The performance is measured with the standard accuracy metric.

The perturbation attacks are carried out only during inference time on both modalities, text and audio. Our general approach for text perturbation is to hide some words by replacing them with the special [MASK] token which BERT uses during pre-training; this technique is also known as *masking*. We perform four text perturbation attacks, which we evaluate independently. The first attack is masking all adjectives—which we recognize as important for emotion recognition—in the transcription.

For control, our second attack is carried out by masking random words, regardless of their part-of-speech category. The number of words we mask equals to the number of adjectives in each utterance. Our third attack focuses on nouns, which typically play a broader semantic role in a sentence. Therefore, we mask all nouns in the input text, and add another attack in which we mask the same number words, chosen randomly ignoring their part-of-speech category. All adjectives and nouns were automatically detected using spaCy.^2^ Their frequencies in the entire corpus are 7.91% for nouns and 3.76% for adjectives.

To perturb the voice signal, we use the librosa audio library (McFee et al., 2015) for modulating the voice fundamental frequency, also perceived as voice pitch, of each utterance by either increasing it or decreasing it by half a tone, chosen randomly. Technically speaking, we use the librosa.effects.pitch_shift procedure by assigning n_steps with either 1 or −1, chosen randomly.

In all our experiments, we fine-tune each model on the original dataset and evaluate it on both versions of the test set, perturbed and unperturbed.

## 5. Results

We report on accuracy values achieved by the three models: text, audio, and multimodal. We evaluate the three models under all perturbation conditions which are relevant for their input modalities. Since the multimodal classifier uses both modalities, we evaluate it on all four perturbed transcriptions, while maintaining their speech input intact, and then on the only one perturbed version of the voice signal, paired along with the original intact transcription. Additionally, we evaluate the multimodal classifier on combinations of each of the four perturbed transcriptions and the pitch-modulated speech signal.

We report on results in four tables. Table 3 summarizes the results attained by the three classifiers on the original, unperturbed inputs. The three other tables include results on perturbed inputs. Tables 3, 4 show the results of the text and audio classifiers, respectively. **Table 6** summarizes the results of the multimodal classifier. Each experiment is executed three times, with each execution running using a different random seed. All accuracy values reported in the four tables are the average of the three different executions.

**Table 3:** Accuracy values achieved by the three classifiers, evaluated on original, unperturbed inputs.

**Model**	**Ses. 1**	**Ses. 2**	**Ses. 3**	**Ses. 4**	**Ses. 5**	**Avg**.
Text	67.44	72.21	63.67	66.40	67.82	67.50
Audio	62.70	60.51	54.76	56.40	57.66	58.40
Multimodal	75.94	79.65	72.63	74.64	76.01	**75.77**

The bold value indicates the best results.

**Table 4:** Accuracy numbers achieved by the text classifier for each IEMOCAP session under different masking conditions.

**Method**	**Ses. 1**	**Ses. 2**	**Ses. 3**	**Ses. 4**	**Ses. 5**	**Avg**.	**Impact**
Baseline	67.44	72.21	63.67	66.40	67.82	67.50	
Adjectives	61.33	68.02	58.69	60.76	61.31	62.02	−8.15%
Random (#adjectives)	62.59	69.06	59.55	61.16	61.60	62.79	−7.01%
Nouns	58.14	63.72	55.92	55.13	58.10	58.20	−13.80%
Random (#nouns)	60.92	65.78	56.94	56.70	59.88	60.04	−11.09%

The Average column has the average accuracy across all sessions. The Impact column measures the difference of the average accuracy in percentage from the Baseline model that analyzes the original unperturbed inputs.

The first row in each of Tables 4–6, represents the baseline experiment, in which we evaluate the models on the original unperturbed input. Essentially, the numbers in the Baseline rows are equal to the numbers appear in the corresponding rows of Table 3. We report on accuracy values achieved by the models on each of the five sessions, while training on the other four sessions as described above. The average of the five sessions is reported in the Avg. column of each table. The Impact column measures in percentage, the difference between the average accuracy and the Baseline average accuracy. Unsurprisingly, all impact values have a negative sign in all our experiments. For convenience reasons, we add another column to Table 6 named *Impact S. Modal* and copy the impact values from the unimodal results tables. The two rows before last in the multimodal table, refer to the experiments we perform with the multimodal classifier processing only one modality (*no text* means using an empty string as input, and *no audio* means using a short audio signal of complete silence). The last row refer to an experiment we perform using unperturbed text, and audio that was augmented with some white noise, generated by random sampling from a zero-mean normal distribution initialized with a standard deviation of 0.1. We discuss the results in the following section.

**Table 5:** Accuracy numbers achieved by the audio classifier for each IEMOCAP session using speech signal whose pitch was intentionally damaged.

**Method**	**Ses. 1**	**Ses. 2**	**Ses. 3**	**Ses. 4**	**Ses. 5**	**Avg**.	**Impact**
Baseline	62.70	60.51	54.76	56.40	57.66	58.40	
Pitch	56.88	53.45	50.55	48.88	51.58	52.26	−10.56%

The Average column has the average accuracy across all sessions. The Impact column measures the difference of the average accuracy in percentage from the Baseline model that analyzes the original unperturbed inputs.

**Table 6:** Accuracy numbers achieved by the multimodal classifier for each IEMOCAP session under different conditions.

**Method**	**Ses. 1**	**Ses. 2**	**Ses. 3**	**Ses. 4**	**Ses. 5**	**Avg**.	**Impact**	**Impact S. Modal**
Baseline	75.94	79.65	72.63	74.64	76.01	75.77		
**Text perturbations**								
Adjectives	71.59	76.13	68.86	69.93	72.67	71.83	−5.20%	−8.15%
Random (#adj.)	72.85	77.11	70.16	70.62	73.15	72.78	−3.95%	−7.01%
Nouns	70.74	73.15	66.47	66.93	69.57	69.37	−8.45%	−13.80%
Random (#noun)	72.10	75.79	68.59	69.75	71.62	71.57	−5.55%	−11.09%
**Audio perturbations**								
Pitch	73.48	78.16	71.33	72.03	73.96	73.79	−2.61%	−10.56%
**Text-audio perturbations**								
Pitch + adj.	68.92	74.98	67.66	67.62	69.87	69.81	−7.88%	−10.56%, −8.15%
Pitch + rand. (#adj.)	70.12	76.04	68.61	68.21	70.42	70.68	−6.73%	−10.56%, −7.01%
Pitch + noun	68.00	71.99	65.12	64.55	66.79	67.29	−11.20%	−10.56%, −13.80%
Pitch + rand. (#noun)	68.79	75.05	67.52	66.84	69.32	69.50	−10.17%	−10.56%, −5.55%
**Eliminating one modality**								
No text	45.90	40.5	46.25	44.12	48.57	45.07	−40.51%	
No audio	60.07	66.63	57.35	64.09	66.96	63.02	−16.82%	
**Adding white noise**								
Noisy audio	54.54	65.41	53.54	67.07	46.69	57.45	−24.17%	

The Avg. (average) column has the average accuracy across all sessions. The Impact column measures the difference of the average accuracy in percentage from the Baseline model, which processed the original unperturbed inputs. In the last column, Impact S.(Single) Modal(ilty) we copy the impact numbers from the Impact columns of Tables 4, 5.

## 6. Discussion

According to the results shown in Table 3, we learn that the multimodal classifier significantly outperforms the two unimodal classifiers. This observation addresses RQ1 and it is aligned with all previous work mentioned above. Additionally, our results show that all the three classifiers perform better on the original unperturbed inputs rather than on their relevant perturbed variation.

Table 6 shows that classifying perturbed inputs makes a larger impact on the two unimodal classifiers than on the multimodal one. The impact made on the text classifier using the adjective-masking attack is −8.15% (±0.12) (i.e., the model performs worse by 8.15% on the perturbed inputs), while the impact on the multimodal classifier is only −5.20% (±1.12). Similarly, the impact made by the noun-masking attack on the text classifier is −13.80% (±1.52), while the impact on the multimodal classifier is only −8.45% (±1.60). Based on these results, we conclude that the multimodal classifier is significantly more resilient to perturbation attacks in which a specific part-of-speech category is masked, than the text classifier is. This conclusion addresses RQ2. We get to the same conclusion for RQ3 by looking at the pitch-modulation attack (audio model: −10.56% ±1.42, multimodal model: −2.61% ±1.37). According to the large (negative) impact values reported on the three last rows of Table 6, we learn that the multimodal classifier performs worse than the unimodal classifiers, either when one modality is completely missing from the input, or when the audio is augmented with some white noise. We find these results to be aligned with Schiappa et al. (2022).

Putting this all together, we find that overall, the multimodal classifier is more robust than the unimodal classifiers, in a way that it can better handle perturbation attacks, designed specifically to either damage or remove information considered important for emotion recognition. Since we perturb the input only at inference time, we believe that this finding means that the multimodal classifier encodes the two modalities in a way that it can use information from one modality to compensate for the other modality, when the latter was flawed. The voice modality helps the model make up for the missing adjectives and nouns, while the transcription helps the model make up for the modulated pitch, even though in both cases, the model was trained on unperturbed instances. The *no text* and *no audio* experiments reinforce this finding, as we learn that when one modality is missing, the moultimodal classifier performs much worse than the unimodal classifiers. Consequently, it shows that during training the multimodal classifier uses information from both modalities, and encodes them in a non-additive way.

In Tables 7, 8, we provide some examples of utterances for which the multimodal classifier returns the correct label even when facing the pitch-modulation attack (in Table 7), and the adjective/noun-masking attacks (Table 8), while the relevant unimodal classifier returns the wrong label.

**Table 7:** Examples of utterances, provided along with classification decisions made by both, the multimodal and the audio classifiers, under pitch-modulation perturbation conditions (low by half tone, high by half tone, and unperturbed).

	**Transcription**	**Label**	**Multimodal**	**Audio**
1	There's people that have given more though, you know?	**sad**		
	*Unperturbed*		**sad** = 0.99, fru < 0.1	**sad** = 0.71, hap = 0.19
	File name: Audio 8.WAV			
	*Pitch-modulated:* ↑*half-tone*		**sad** = 0.99, fru < 0.1	**fru = 0.70**, hap = 0.23
	File name: Audio 6.WAV			
	*Pitch-modulated:* ↓*half-tone*		**sad** = 0.99, hap < 0.1	**hap** = 0.56, fru = 0.31
	File name: Audio 7.WAV			
2	Oh. Totally.	**hap**		
	*Unperturbed*		**hap** = 0.98, fru = 0.01	**hap** = 0.46, fru = 0.34
	File name: Audio 5.WAV			
	*Pitch-modulated:* ↑*half-tone*		**hap** = 0.99, fru < 0.1	**ang** = 0.96, fru = 0.02
	File name: Audio 3.WAV			
	*Pitch-modulated:* ↓*half-tone*		**hap** = 0.99, fru < 0.1	**ang** = 0.74, fru = 0.23
	File name: Audio 4.WAV			

For each example, we provide the two labels with the highest probability, as generated by each model (the final label is in boldface). For reference, we provide the audio files in the Supplementary material (the files can be located with the file name mentioned under each utterance). The true label is provided in the Label column.

**Table 8:** Examples of utterances, provided along with classification decisions made by both, the multimodal and the text classifiers, under the adjective- and noun-masking attacks.

	**Transcription**	**Label**	**Multimodal**	**Text**
1	*Unperturbed*			
	I waited the entire baggage- I waited for the whole	**fru**	**fru** = 0.99, ang < 0.1	**fru** = 0.80, ang < 0.1
	baggage carousel four times. They told me to go to			
	the next one. I waited through that for three planes			
	and now I'm here.			
	**File name:** Audio 2.WAV			
	*Adjective-masking*			
	I waited the [MASK] baggage- I waited for the [MASK]		**fru** = 0.88, ang = 0.11	**fru** = 0.52, sad = 0.38
	baggage carousel four times. They told me to go to			
	the [MASK] one. I waited through that for three planes			
	and now I'm here.			
	*Noun-masking*			
	I waited the entire [MASK]- I waited for the whole		**fru** = 0.63, ang < 0.1	**hap** = 0.49, fru = 0.29
	[MASK] [MASK] four [MASK]. They told me to go to			
	the next [MASK]. I waited through that for three [MASK]			
	and now I'm here.			
2	*Unperturbed*			
	Then why is she still single? New York is full	**ang**	**ang** = 0.99, fru < 0.1	**ang** = 0.45, fru = 0.31
	of men, why is she still single? Probably a hundred			
	people told her she's foolish, but she waited.			
	**File name:** Audio 1.WAV			
	*Adjective-masking*			
	Then why is she still [MASK]? New York is [MASK]		**ang** = 0.81, hap = 0.16	**hap** = 0.69, fru = 0.19
	of men, why is she still [MASK]? Probably a hundred			
	people told her she's [MASK], but she waited.			
	*Noun-masking*			
	Then why is she still single? New York is full		**ang** = 0.99, fru < 0.1	**fru** = 0.63, hap = 0.17
	of [MASK], why is she still single? Probably a hundred			
	[MASK] told her she's foolish, but she waited.			

For each example, we provide the two labels with the highest probability, as generated by each model (the final label is in boldface). For reference, we provide the audio files in the Supplementary material (the files can be located with the file name mentioned under the unperturbed version of each utterance). The true label is provided in the Label column.

Interestingly enough, we see some indications that the transcription is more important than the voice for multimodal emotion recognition. Since the pitch-modulation attack makes a relatively small impact on the multimodal classifier, we assume that the text, and the way that it is encoded with BERT, contains more useful information than voice for classification. However, it could also be attributed to other causes, such as inefficient voice attacks, or the way voice is encoded by wav2vec 2.0.

Looking at the results reported in Table 4, we realize that masking random words makes a smaller impact on the text model than masking a specific part-of-speech category. This result is repeated in the multimodal analysis. Since the number of random words we choose to mask is equal to the number of words of the specific category we mask, the results suggest that adjectives and nouns are more important for emotion recognition than a randomly chosen set of words. It seems like nouns make a bigger impact on both, text and multimodal models. However, it is hard to conclude that nouns are more important for emotion recognition than adjectives, since nouns are more frequent than adjectives in our corpus (7.91% vs. 3.76%). More work is needed in order to compare the importance of specific part-of-speech categories for emotion recognition.

Combining perturbation attacks on both modalities, results in impact values equivalent to the sum of the impact values made by the two individual attacks (combining pitch and adjectives: −7.88% vs. sum of individual attacks: −7.81%; combining pitch and nouns: −11.20% vs. sum of individual attacks: −11.06%). It shows that the multimodal classifier remains resilient to the same adjective- and noun-masking attacks, even when the pitch is changed. Similarly, multimodal model remains resilient to the pitch-modulation attack even when either adjectives or nouns are gone.

## 7. Limitations

In this study we have used the IEMOCAP dataset, a collection of recordings of five dyadic sessions of different pairs of actors, one female and one male, all speak English. The topics discussed in the recordings are selected by the actors. Therefore, the conclusions drawn in this paper are limited to English and to the specific discussed topics. Additionally, our methodology includes using wav2vec 2.0 and BERT, which may be biased toward the dataset that they have been trained on.

## 8. Conclusions

In this work, we measure the robustness of a multimodal audio-text classifier for emotion recognition, by intentionally perturbing one modality of the input and monitoring the change in classification performance. The results of all our experiments show that the multimodal classifier is more resilient to perturbation attacks designed specifically for emotion recognition, than the corresponding unimodal classifiers. The perturbation attacks, which we use in this study, are designed to hide adjectives and nouns from the transcriptions, as well as to modulate the voice pitch of the speech signal. Based on the results of this work, we recommend to include the transcription of the speech as an input for an audio-based emotion recognition system. However, more work is still needed to evaluate some additional types of attacks, which are not necessarily designed for emotion recognition. Extending this study to evaluate other relevant modalities, is one of our future directions.

## Data availability statement

Publicly available datasets were analyzed in this study. This data can be found here: https://sail.usc.edu/iemocap/index.html.

## Author contributions

DC and IR coded the algorithms and executed the experiments. KB and MB wrote the discussion and conclusions sections. All authors contributed to the conception, design, and analysis of the study and experiments. All authors contributed to the article and approved the submitted version.

## Footnotes

^1^Specifically, we use the bert-base-uncased model of Hugging Face (Wolf et al., 2019).

^2^ https://spacy.io
